# Hospital Schools for Domestics

**Published:** 1912-08-10

**Authors:** 


### INSTITUTIONAL HOUSEKEEPING.

### Hospital Schools for Domestics.

Owing to the great difficulty of maintaining the domestic staff at its full number, the Matron of a provincial Hospital, about four years ago, commenced, with the consent of her Committee, the experiment of taking young girls of sixteen years of age to train in the " domestic service of the institution." It had long been the custom to employ young girls, who lived in, as wardmaids, but only the most inefficient help had been available, and the maids were perpetually changing. Since the institution of the training system there has been a marked improvement, and a far better class of maid is now employed, while the length of service covers two years and more in many cases.

Printed forms of rules, which are of the simplest, and a form of application asking a few equally simple questions are given to the candidates, who, if accepted, are taken for a month on trial. At the end of a month's trial, if found satisfactory the candidate is appointed as wardmaid and promises to stay for two years. She is then given material for uniform, and takes a junior place in the establishment.

The terms of service require that candidates are in good health, can be well recommended, know something of housework and plain sewing, and can write neatly. The consent of parents is required.

Material for three dresses and six aprons is given each year. An increase of salary is given during the second year, and if the full term of two years' training is satisfactorily completed, a bonus of £2 and a testimonial (for which a special form is used) is given.

Off-duty time is given daily, extra time on Sundays, and on one day in the week, a day off each quarter, and a holiday at the end of the first year. Off-duty time is given to the young maids before 6.30 p.m. and not in the late evenings.

At the end of the two years' training the maids are eligible to take the better posts, as under housemaids, or dormitory or dining-room maids, when they receive an advance in salary, and wear a slightly different dress, only noticeable amongst themselves, but meaning promotion.

A needlework class is held weekly, and in winter a choir practice.

The commencement of such a plan requires effort and co-operation on the part of the matron and her assistant, but it is well worth while.

There is every reason to believe that if many hospitals would work on similar lines a new opening would be made for the many respectable girls who now drift either into ill-paid work in cheap shops, or who become " day-girls " and learn nothing properly. To go out to service has not much attraction for them, but the thought of entering a hospital to train in " domestic service " puts the work in a different light. Having entered, the maids rise to the position. They can be taught that the humbler work for which they are so well fitted is only part of a great whole. Their sympathies can be aroused and a spirit of *esprit de corps* inculcated. To have this feeling amongst the domestic staff largely assists in the smooth working of the establishment.

At an age when character is being formed young girls are peculiarly sensitive to influence for good. The constant association with those who work for the sick, the example of devotion to duty set by a good Ward Sister and conscientious Nurses, the kindly interest in their personal concerns shown by the Home Sister, all tend to help them to do well, carrying as they do an incentive to follow in the same steps.

The Matron, who, with some misgiving, commenced working on these lines, has already had much encouragement, and is very hopeful for the future.

### Housekeeping Queries.

#### How to Admit Air without Light.

A. E., Coventry.—The problem of introducing plenty of fresh air into bedrooms at night without noise or light is a very important one. The best way to solve it is to have outside jalousies, as they do in foreign countries, with wide slats through which the air percolates freely. All nursing homes and night nurses' quarters should be fitted with them. Venetian blinds are unsatisfactory on account of the rattling, and the ordinary blinds with lath and acorn would, as you say, keep the soundest sleeper awake on a windy night. Try fitting curtains made of dark-blue casement cloth, lined with buff, across the bow and the smaller windows. They should reach down about 18 inches below the bottom of the window, so that they can be blown out freely without admitting sunlight or moonlight. They must be hung on smallish rings sewn very close together, and the rod should be fitted on to a wide window board, with deep valance, projecting a foot beyond the top of the window on either side. Great care must be taken to fasten each curtain securely close to the wall at the far end, so that no slits of light penetrate when they are drawn. The curtains should be made wide enough to fold securely over each other in the middle, and safety pins should be used to prevent any disarrangement in case the wind gets up in the night. If this plan is carried out intelligently the room will be free from any but subdued light, even on an east aspect, and the sounds made by the moving curtain will be equally subdued.

#### Unpolished Rice.

Novice.—There is no doubt that rice loses some valuable property in the polishing process practised to prepare it for the market. The best stores will always supply unpolished rice if requested, and it is to be preferred. The proportion of nitrogenous matter (gluten) in rice is less than in other cereals, only 7 per cent., as compared with 22 per cent. in the finest wheat and 14 per cent. in oats, so that it is the more important to retain all its goodness.

NOTICE TO MATRONS.

We are always pleased to answer queries and give recipes which may be asked for by our readers. Let us know if any difficulties are hampering you, and we will do our best to find a remedy. Any successful experiments you may be making in culinary matters would interest a wide circle.

